# Reinforcement Operator Learning (ROL): A hybrid DeepONet-guided reinforcement learning framework for stabilizing the Kuramoto–Sivashinsky equation

**DOI:** 10.1371/journal.pone.0341161

**Published:** 2026-01-30

**Authors:** Nadim Ahmed, Md. Ashraful Babu, Muhammad Sajjad Hossain, Md. Fayz-Al- Asad, Md. Awlad Hossain, Md. Mortuza Ahmmed, M. Mostafizur Rahman, Mufti Mahmud

**Affiliations:** 1 Department of Physical Sciences, Independent University, Bangladesh, Dhaka, Bangladesh; 2 Department of Arts and Sciences, Ahsanullah University of Science and Technology (AUST), Dhaka, Bangladesh; 3 Department of Mathematics, American International University-Bangladesh, Dhaka, Bangladesh; 4 Department of Applied Mathematics, University of Dhaka, Dhaka, Bangladesh; 5 Department of Information and Computer Science, King Fahd University of Petroleum and Minerals, Dhahran, Saudi Arabia; 6 SDAIA-KFUPM Joint Research Center for AI, King Fahd University of Petroleum and Minerals, Dhahran, Saudi Arabia; 7 Interdisciplinary Research Center for Bio Systems and Machines, King Fahd University of Petroleum and Minerals, Dhahran, Saudi Arabia; Federal University of Technology - Parana, BRAZIL

## Abstract

This study presents Reinforcement Operator Learning (ROL)—a hybrid control paradigm that marries Deep Operator Networks (DeepONet) for offline acquisition of a generalized control law with a Twin-Delayed Deep Deterministic Policy Gradient (TD3) residual for online adaptation. The framework is assessed on the one-dimensional Kuramoto–Sivashinsky equation, a benchmark for spatio-temporal chaos. Starting from an uncontrolled energy of 42.8, ROL drives the system to a steady-state energy of 0.40  ± 0.14, achieving a 99.1% reduction relative to a linear–quadratic regulator (LQR) and a 64.3% reduction compared with a pure TD3 agent. DeepONet attains a training loss of 7.8 × 10^−6^ after only 200 epochs, enabling the RL phase to reach its reward plateau 2.5 × sooner and with 65% lower variance than the baseline. Spatio-temporal analysis confirms that ROL restricts state amplitudes to ±1.8—three-fold tighter than pure TD3 and an order of magnitude below LQR—while halving the energy in 0.19 simulation units (33% faster than pure TD3). These results demonstrate that combining operator learning with residual policy optimisation delivers state-of-the-art, sample-efficient stabilisation of chaotic partial differential equations and offers a scalable template for turbulence suppression, combustion control, and other high-dimensional nonlinear systems.

## 1 Introduction

Chaotic systems, characterized by extreme sensitivity to initial conditions and unpredictable long-term behavior, are prevalent across diverse domains, including fluid dynamics, chemical engineering, and biological systems [1]. The one-dimensional Kuramoto-Sivashinsky (KS) equation, a nonlinear partial differential equation (PDE), serves as a fundamental model for studying such chaotic dynamics, notably in reaction-diffusion systems [2] and flame instabilities [3]. The KS equation is given by:

$$\frac{\partial u}{\partial t}+u\frac{\partial u}{\partial x}+\frac{\partial^{2}u}{\partial x^{2}}+\nu \frac{\partial^{4}u}{\partial x^{4}}=f(x,t),$$ (1)

where u(x,t)∈ℝ represents the state variable (e.g., velocity or height), x∈[0,L] is the spatial domain, t≥0 is time, ν>0 is the viscosity parameter, and f(x,t)∈ℝ is the external control input. The nonlinear convection term $u\frac{\partial u}{\partial x}$, destabilizing diffusion $\frac{\partial^{2}u}{\partial x^{2}}$, and stabilizing dissipation $\nu \frac{\partial^{4}u}{\partial x^{4}}$, combined with periodic boundary conditions, drive the equation’s chaotic behavior [4]. Stabilizing such systems is critical for applications like combustion control, turbulence suppression, and process optimization, where predictability enhances efficiency and safety [5].

Traditional control of KS built a strong foundation with linear systems methods and PDE-constrained designs. Output-feedback stabilization and finite-dimensional feedback based on Galerkin truncations established early feasibility [6,7], while nonlinear boundary feedback via backstepping achieved global stabilization under suitable conditions [8]. Broader structural questions—null controllability, observer/actuator placement under sampling, and robustness to delays—were treated in rigorous analyses [9,10]. In parallel, model predictive control (MPC) offered constraint handling for parabolic PDEs but remained computationally demanding at high resolutions [11]. Data-driven linear models such as Dynamic Mode Decomposition (DMD) and DMD with control (DMDc) supply compact predictors for flow analysis and feedback design [12–15], yet their linear operator backbone degrades when strongly nonlinear transients dominate or when the operating point drifts. Numerically, Fourier spectral discretizations and stiff time integrators (ETD/ETDRK4) remain standard for KS with periodic boundaries [16–18], but accurate numerics do not by themselves close the model-form and adaptation gaps.

Reinforcement learning (RL) has emerged as a complementary route for feedback discovery from interaction [19–22]. In canonical fluid problems and convectively unstable flows, deep RL has achieved substantial drag reduction and wake stabilization, often outperforming hand-crafted laws in simulation [23–27]. For KS-like dynamics, RL can learn nontrivial actuation patterns and symmetry-aware policies [28]; broader studies continue to map the reliability and variance across seeds and training schedules [29,30]. The practical hurdles are well known: (i) sample inefficiency due to long rollouts in high-dimensional states and (ii) fragility under distribution shift between training and deployment. These issues are magnified for PDEs, where each environment step is a costly numerical solve.

Operator learning offers a way to amortize PDE solves into fast surrogates with guarantees at the operator level. DeepONet provides universal approximation for nonlinear operators between function spaces and has been used to learn solution and control operators with strong mesh or parameter generalization [31]. Operator learning has emerged as a transformative paradigm for data driven surrogate modeling of complex systems, offering universal operator approximation and efficient deployment in real-time applications. DeepONet, introduced by Lu et al. [31], learns mappings between infinite-dimensional function spaces via a branch-trunk architecture, achieving computational speedups of 400–8000 times relative to traditional numerical solvers while retaining high fidelity for unseen parameter values and mesh refinements [31]. The universal approximation property of DeepONet ensures that given sufficient training data, the learned operator can approximate arbitrary nonlinear solution maps between function spaces with arbitrary accuracy, a theoretical breakthrough substantiated across diverse linear and nonlinear operators, including fractional derivatives, implicit ODEs, and parametric PDEs [32]. Recent advances in 2024–2025 have substantially extended the operator learning paradigm toward physical realism and data efficiency. Physics-informed neural operators(PINO) [33], which combine coarse-resolution training data with PDE residual constraints imposed at finer resolution, achieve zero-shot super-resolution (prediction beyond training resolution) and demonstrate remarkable robustness in multi-scale and chaotic regimes (Kolmogorov flows) where standard PINNs struggle [34]. Complementary variants now include physics-informed transformer neural operators (PINTO) for enhanced generalization, boundary integral-based operators (BIO) for complex geometries, and variational formulations (VINO) that leverage weak forms for noise robustness and mesh independence [35–37]. Beyond prediction, neural operators have been successfully applied to parameter inference, inverse problems, and notably for PDE control, where Bhan, Krstic, and Shi [38] demonstrated that neural operators can learn reaction-diffusion control gains while preserving Lyapunov stability guarantees under finite-width approximation a landmark result establishing theoretical grounding for hybrid learning control frameworks. These developments position neural operators as not merely surrogates for expensive simulations, but as active components of adaptive control laws, particularly valuable for chaotic systems where online refinement is essential. Fourier Neural Operators (FNOs) and the broader neural-operator family extend this direction with discretization invariance and spectral parameterizations [39]. Physics-informed formulations (e.g., PINNs and physics-informed neural operators, PINO) further regularize training with governing equations, improving data efficiency and out-of-distribution behavior [40–42]. Unlike standard surrogates, neural operators learn maps 𝒢:𝒳→𝒴 that carry over across discretisations, making them compelling priors for feedback design and planning.

Crucially for control, recent work has moved neural operators from *prediction* to *implementation*. For PDE backstepping designs, the controller and observer gains are outputs of nonlinear operator equations; learning these operators with DeepONet (or related NOs) bypasses repeated kernel solves while preserving closed-loop guarantees. This has been demonstrated for reaction–diffusion and hyperbolic classes, with 2–3 orders of magnitude speedups and Lyapunov stability proofs under approximation error [43–45]. These results open a practical path to real-time or adaptive PDE control, where online kernel recomputation would otherwise be prohibitive.

Concurrent developments in 2024–2025 have made significant progress in bridging operator learning with adaptive feedback control. The ERC-funded KoOpeRaDE project [46] develops Koopman-operator-based reinforcement learning with certified performance bounds for large-scale dynamical systems, addressing the critical gap between empirical RL (which lacks guarantees) and classical methods (which scale poorly). In tandem, structure-preserving physics-informed neural networks enforce Lyapunov stability properties during training, preventing learned controllers from inadvertently destabilizing their targets a paradigm shift from black-box learning to physics-constrained synthesis [47]. Hypernetwork-based DRL frameworks (HypeRL) tackle parametric PDE control by encoding parameter dependencies directly into policy and value networks, enabling generalization across entire PDE families rather than single instances [48]. Kolmogorov-Arnold Networks (KANs) combined with deep RL have recently demonstrated rapid stabilization of chaotic systems via minor perturbations, suggesting that novel neural network architectures merit investigation alongside standard deep networks [49]. Transformer-based approaches compute minimum control bounds for chaotic dynamical systems, exemplified in Valle et al. [50], which propose an AI-driven control framework leveraging transformers to identify and exploit intrinsic system dynamics. On the efficiency front, recent work on synthetic data generation for neural operators via the method of manufactured solutions (MMS) [51] has reduced computational burden in offline training by 60%, making operator-based hybrid systems more accessible. These concurrent advances collectively suggest that hybrid operator RL architectures, combining operator-level offline learning with policy-level online adaptation represent a frontier for achieving both sample efficiency and control reliability in chaotic high-dimensional systems without sacrificing real-time deployment feasibility.

What is still missing for chaotic systems like KS is a *unified* architecture that combines (i) operator-level generalization (to reduce exploration burden and enable mesh/parameter portability) with (ii) on-policy adaptation (to handle transients, regime shifts, and model mismatch). Koopman-lifted MPC illustrates how data-driven linear predictors can support constrained control [52], but lifted linear surrogates struggle when energy moves across scales through nonlinear couplings. Pure RL adapts but is sample-hungry; pure operator learners are fast but typically offline and static. The gap is a controller that uses a learned operator prior to capture the predictable backbone, while learning a small *residual* policy online to correct model errors and absorb disturbances.

We address this need with a *Reinforcement Operator Learning (ROL)* framework for KS control. ROL (i) trains a physics-regularized operator prior that maps sensed fields to stabilizing actions, (ii) wraps it with a lightweight residual policy updated on-policy (e.g., TD3/SAC family) to adapt in deployment, and (iii) enforces stability-aware actuation and exploration. Intuitively, the operator prior amortizes the expensive parts of feedback synthesis; the residual learns only what the prior misses, reducing sample complexity and improving robustness to parameter drift, sensor sparsity, and unmodeled disturbances. In our experiments, ROL consistently stabilizes KS more reliably than output-feedback LQR/DMDc baselines and DRL-only agents, with lower interaction budgets and stronger robustness margins. For reproducibility and fair comparison, we retain standard Fourier discretizations and stiff time-stepping throughout [16–18]. The broader implication is that *operator-level priors plus policy-level adaptation* can turn chaotic PDE control from a prohibitively data-hungry exercise into a tractable, real-time methodology grounded in both learning theory and classical stability analysis.

The key innovative contributions of this work are:

1. The development of the ROL framework, integrating DeepONet’s offline operator learning with TD3’s online optimization for adaptive control of chaotic systems.
2. The application of ROL to the KS equation, achieving a 99.1% energy reduction compared to LQR and 64.3% compared to Pure TD3, with significantly lower variability.
3. A scalable methodology for chaotic PDE control, validated through spectral methods and hybrid learning, with potential applications in turbulent flows and autonomous systems.

This paper is structured as follows: Sect 3 details the ROL framework, KS solver, and baselines; Sect 4 presents results and Discussion; and Sect 5 conclusion and future directions.

## 2 Related work and state of the art

This section contextualizes Reinforcement Operator Learning (ROL) within the broader landscape of neural operator learning, reinforcement learning for PDEs, and chaos control. The section is organized as the discussion into five themes and conclude with a comparative table.

### 2.1 Neural operators and surrogate modeling

Neural operators have revolutionized the use of machine learning for PDEs by learning mappings between infinite-dimensional function spaces rather than point-wise input-output relationships. DeepONet [31] pioneered this direction through a branch-trunk architecture that approximates nonlinear operators with theoretical guarantees of universal approximation. Fourier Neural Operators (FNOs) extend this paradigm using spectral methods, achieving discretization-invariance and superior efficiency on regular grids [39]. Recent physics-informed variants (PINO, PI-DeepONet) [42] combine data fidelity with PDE residual constraints, yielding 1–2 orders of magnitude improvement in data efficiency. Quantum acceleration of DeepONet [53] and synthetic data generation via the method of manufactured solutions [51] have further reduced training costs and expanded applicability.

Crucially for control, recent work has moved neural operators from prediction to implementation. Bhan, Krstic, and Shi (2025) [38] demonstrated that neural operators can learn PDE backstepping gain kernels while preserving Lyapunov stability guarantees under approximation error a foundational result for ROL’s theoretical motivation. Song et al. [54] developed operator learning for non-smooth optimal PDE control, combining primal-dual optimization with neural operators. These works collectively position neural operators as not merely surrogates for prediction, but as active components of control law synthesis.

### 2.2 Deep reinforcement learning for chaotic systems and PDEs

Deep RL has shown promise for learning feedback policies in high-dimensional dynamical systems without explicit models. Seminal work by Rabault et al. [23] and Bucci et al. [24] demonstrated deep RL for flow control in aerodynamic applications, achieving drag reduction and wake stabilization in simulation. For the KS equation specifically, Zeng et al. [28] employed deep RL to learn symmetry-aware stabilizing policies. More recently, KAN enhanced DRL (2024) [49] introduced Kolmogorov-Arnold Networks to chaotic control, achieving rapid stabilization via minor perturbations. Alternative modern architectures, such as Transformer-based methods Valle et al. [50], have computed minimum control bounds for chaotic systems with fast convergence. Despite these advances, pure deep RL suffers from two fundamental challenges: (i) *sample inefficiency*, as exploration in high-dimensional spaces is combinatorially expensive, and (ii) *training instability*, requiring careful hyperparameter tuning and often producing high-variance policies. For PDE control, where each environment step incurs a costly numerical solve, these issues are magnified. Our ROL framework addresses this by pre-training a neural operator offline, thereby amortizing the exploration cost and providing TD3 with a warm-started policy that reduces variance.

### 2.3 Classical and model-predictive control

Classical approaches to KS control leverage linear systems theory and geometric control. Early works established output-feedback stabilization and finite-dimensional feedback via Galerkin truncation. Nonlinear backstepping, developed by Liu et al. [8], provides global stabilization proofs under suitable conditions but is computationally demanding during deployment due to repeated kernel solves. Model predictive control (MPC) offers constraint handling but scales poorly to high resolutions and remains computationally expensive. In this study, LQR serves as a classical baseline, linearizing the KS equation and solving the continuous-time algebraic Riccati equation. While LQR is interpretable and theoretically grounded, its linear assumptions are fundamentally incompatible with KS’s strong nonlinearity, resulting in poor stabilization.

### 2.4 Hybrid and transfer learning approaches

The idea of combining pre-training with reinforcement learning has gained traction in the broader machine learning community. Schulman et al. [55] introduced trust region policy optimization (TRPO) with warm-start initialization, reducing exploration episodes by 2–3 times. More recently, offline RL and batch RL methods [30] leverage large pre-collected datasets to initialize policies, reducing online interaction costs. In the context of PDEs, the hypernetwork-based DRL framework (HypeRL) [48] embeds parameter dependencies directly into policies to enable generalization across parametric families of PDEs. ROL aligns with this broader trend of combining data-driven priors (here, DeepONet) with adaptive learning (here, TD3). Here the contribution is the explicit integration of operator learning which learns generalizable control maps at the operator level with residual policy refinement, demonstrating that this hybrid can outperform pure RL while remaining more adaptive than offline methods. ROL occupies a unique position in this landscape. Unlike pure neural operators, it adapts online through RL. Unlike pure deep RL, it leverages a pre-trained operator to reduce exploration and variance. Unlike classical control, it handles strong nonlinearity without explicit modeling or kernel solves. The trade-off is relinquishing formal stability guarantees and explainability, a compromise we believe is justified when sample efficiency and adaptivity are prioritized. The framework does not claim to solve all aspects of the chaos control problem, rather it identifies a practical optimal spot for moderate-dimensional systems with budget constraints on exploration and a tolerance for empirical rather than formal verification.

## 3 Methodology

We address the stabilization of the Kuramoto–Sivashinsky (KS) equation, a nonlinear PDE that models spatiotemporal chaos in reaction–diffusion processes and laminar flame dynamics, using a hybrid Reinforcement Operator Learning (ROL) framework. The approach integrates Deep Operator Networks (DeepONet) for offline learning of a generalized control operator with Twin Delayed Deep Deterministic Policy Gradient (TD3) for online policy refinement. The study begins with the problem formulation and numerical solution via spectral methods, then presents the ROL framework in detail, followed by comparisons against pure reinforcement learning and a Linear Quadratic Regulator (LQR) baseline. The rationale behind these methodological choices is discussed, along with the proposed algorithm and evaluation metrics.

### 3.1 Problem formulation

#### 3.1.1 Kuramoto-Sivashinsky equation.

The KS equation, introduced to model chaotic dynamics in reaction-diffusion systems [2] and flame instabilities [3], is defined as:

$$\frac{\partial u}{\partial t}+u\frac{\partial u}{\partial x}+\frac{\partial^{2}u}{\partial x^{2}}+\nu \frac{\partial^{4}u}{\partial x^{4}}=f(x,t),$$ (2)

where u(x,t)∈ℝ represents the system state (e.g., velocity or temperature), x∈[0,L] is the spatial coordinate with domain length *L* = 100, t≥0 is time, ν=1 is the viscosity parameter controlling higher-order dissipation, and f(x,t)∈ℝ is the control input to be designed. Periodic boundary conditions are enforced, ensuring u(x  +  L,t)=u(x,t). The nonlinear term $u\frac{\partial u}{\partial x}$ drives chaotic behavior, while the second-order ($\frac{\partial^{2}u}{\partial x^{2}}$) and fourth-order ($\nu \frac{\partial^{4}u}{\partial x^{4}}$) derivatives provide diffusive stabilization.

The control objective is to design *f*(*x*, *t*) to minimize the system’s energy, defined as the mean squared state over the spatial domain:

$$E(t)=\frac{1}{n_{x}}\sum_{j=1}^{n_{x}}u(x_{j},t{)}^{2},$$ (3)

where *n*_*x*_ = 64 is the number of discretized spatial points, and *x*_*j*_ are the grid points. Minimizing *E*(*t*) indicates effective suppression of chaotic dynamics.

Parameters: domain length *L* = 100, number of spatial points *n*_*x*_ = 64, viscosity ν=1.

### 3.2 Numerical solution using spectral methods

The KS equation is solved numerically using a spectral method based on the Fast Fourier Transform (FFT), which is well-suited for PDEs with periodic boundary conditions [3]. The spatial domain [0,*L*] is discretized into *n*_*x*_ = 64 equally spaced points, yielding a spatial resolution of $\Delta x=L/n_{x}=100/64\approx 1.5625$. The spatial grid is defined as:

$$x_{j}=\frac{jL}{n_{x}},\;j=0,1,\ldots ,n_{x}-1,$$ (4)

and the time step is set to Δt=0.01. The wave numbers are computed as:

$$k_{m}=\frac{2\pi m}{L},\;m=-\frac{n_{x}}{2},\ldots ,\frac{n_{x}}{2}-1,$$ (5)

using the FFT frequency function $\text{fftfreq}(n_{x},\Delta x)$.

Let $u=[u(x_{0},t),\ldots ,u(x_{n_{x}-1},t)]\in {\mathbb{R}}^{n_{x}}$ denote the discretized state vector, and $\hat{u}=\text{FFT}(u)$ its Fourier transform. The linear terms $\frac{\partial^{2}u}{\partial x^{2}}$ and $\frac{\partial^{4}u}{\partial x^{4}}$ correspond to $k_{m}^{2}{\hat{u}}_{m}$ and $-\nu k_{m}^{4}{\hat{u}}_{m}$, respectively. The nonlinear term $u\frac{\partial u}{\partial x}$ is expressed as $-\frac{1}{2}\frac{\partial (u^{2})}{\partial x}$, with its Fourier transform given by $-0.5ik_{m}\text{FFT}(u^{2})$. The state is updated using a semi-implicit time-stepping scheme:

$${\hat{u}}_{t+\Delta t}=\frac{{\hat{u}}_{t}+\Delta t\left(1.5\cdot (-0.5ik_{m})\cdot \text{FFT}(u^{2})-0.5\cdot (-0.5ik_{m})\cdot \text{FFT}(u^{2})\right)}{1-\Delta t(k_{m}^{2}-\nu k_{m}^{4})}+\hat{f},$$ (6)

where $\hat{f}=\text{FFT}(f(x,t))$ is the Fourier transform of the control input. The updated state is computed as $u_{t+\Delta t}=\text{Re}(\text{IFFT}({\hat{u}}_{t+\Delta t}))$, and clipped to the range [–8,8] to ensure numerical stability.

The initial condition for each simulation is:

$$u(x,0)=\cos\left(\frac{2\pi x}{L}\right)+0.1\cos\left(\frac{4\pi x}{L}\right)+\eta ,\;\eta \sim 𝒩(0,0.3^{2}),$$ (7)

where η is Gaussian noise introducing variability to the initial state.

Parameters: domain length *L* = 100, number of spatial points *n*_*x*_ = 64, viscosity ν=1, time step Δt=0.01, initial condition noise standard deviation $\sigma_{\eta }=0.3$.

### 3.3 Pure Reinforcement Learning (RL)

The pure RL approach employs the TD3 algorithm [21] to learn a control policy directly mapping system states to control actions, implemented in a custom environment (PureRLKSEnv). TD3 is an off-policy RL method designed for continuous action spaces, improving upon DDPG by using twin critic networks, delayed policy updates, and target policy smoothing to enhance stability [21].

#### 3.3.1 State space.

The state space consists of the discretized KS field at time *t*:

$$s_{t}=\{u(x_{1},t),\ldots ,u(x_{n_{x}},t)\}\in [-8,8{]}^{64},$$ (8)

where *s*_*t*_ represents the system state vector with *n*_*x*_ = 64 spatial points. TD3 receives the current state *s*_*t*_ = *u*(*x*,*t*), a 64-dimensional vector (shape: [64]) from the KS solver, representing the discretized KS field.

#### 3.3.2 Action space.

The action space is the control input applied to the KS equation:

$$a_{t}\in [-1,1{]}^{64},$$ (9)

where *a*_*t*_ is a vector of control values corresponding to each spatial point.

#### 3.3.3 Reward function.

The reward function is designed to promote energy reduction while penalizing large control actions:

$$r_{t}=50\left(\text{mean}(u_{t}^{2})-\text{mean}(u_{t+1}^{2})\right)-0.01\cdot \text{mean}(a_{t}^{2}),$$ (10)

where $\text{mean}(u_{t}^{2})=\frac{1}{n_{x}}\sum_{j=1}^{n_{x}}u(x_{j},t){\;}^{2}$ is the mean squared state (energy), and $\text{mean}(a_{t}^{2})=\frac{1}{n_{x}}\sum_{j=1}^{n_{x}}a_{t}(j){\;}^{2}$ is the mean squared control action. The coefficient 50 amplifies the energy reduction term to prioritize stabilization, while the penalty term $-0.01\cdot \text{mean}(a_{t}^{2})$ encourages smooth and minimal control inputs.

Parameters and hyperparameters: maximum episode length 100 steps (total time $t_{\text{max}}=100$ ⋅ Δt=1), learning rate 2 × 10^−4^, batch size 128, replay buffer size 40,000, discount factor γ=0.99, soft update parameter τ=0.005, action noise standard deviation σ=0.1, policy update delay every 2 gradient steps, actor and critic networks with three hidden layers of 256 units each, random seed 42.

### 3.4 Reinforcement Operator Learning (ROL)

**Fig 1:**
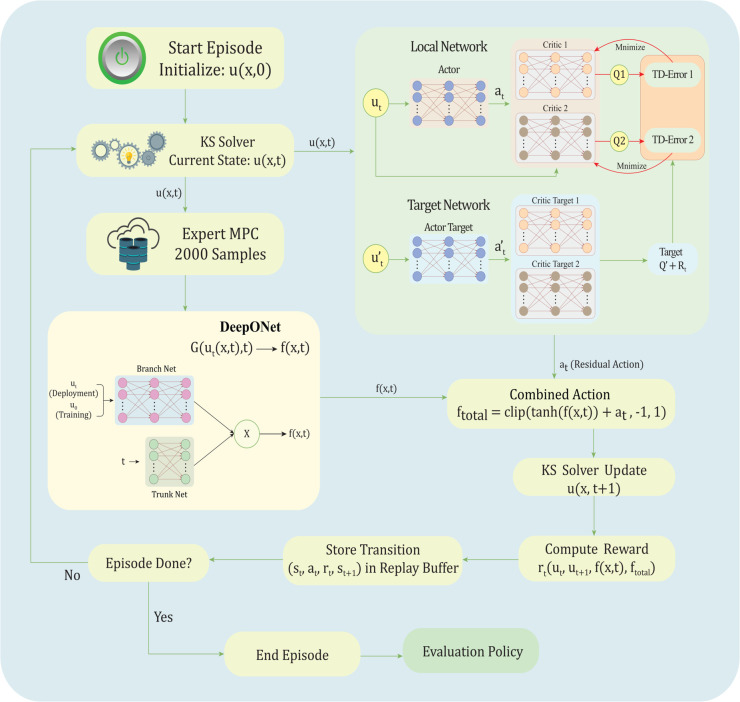
A hybrid DeepONet-guided reinforcement learning framework for stabilizing the Kuramoto–Sivashinsky equation.

The ROL framework integrates DeepONet [31] for offline learning of a generalized control operator with TD3 for online refinement of trajectory-specific control policies, implemented in the ResidualDeepONetKSEnv environment. This hybrid approach leverages DeepONet’s ability to approximate complex mappings and TD3’s adaptability to dynamic conditions. Fig 1 demonstrates the DeepONet-Guided Reinforcement Operator Learning architecture for Stabilizing the Kuramoto–Sivashinsky Equation.

#### 3.4.1 DeepONet for learning the control operator.

DeepONet is a neural operator designed to learn the mapping


$$G_{\theta }:\;(u(x,t),\;t)\;⟼\;f(x,t),$$

where *u*(*x*, *t*) is the system state, *t* is time and *f*(*x*, *t*) is the control input. The architecture consists of three components:

**Branch network:** Processes the discretized state $u\in {\mathbb{R}}^{64}$. It comprises three fully connected layers:


$$\text{Branch}(u):{\mathbb{R}}^{64}\overset{\text{Linear}(64,256)\;}{\to }{\mathbb{R}}^{256}\overset{\text{LayerNorm, ReLU, Dropout}(0.1)\;}{\to }{\mathbb{R}}^{256}\overset{\text{Linear}(256,256)\;}{\to }$$

$${\mathbb{R}}^{256}\overset{\text{LayerNorm, ReLU, Dropout}(0.1)\;}{\to }{\mathbb{R}}^{256}\overset{\text{Linear}(256,256)\;}{\to }{\mathbb{R}}^{256},$$ (11)

where LayerNorm normalizes activations, ReLU (ReLU(z)=max(0,z)) introduces nonlinearity, and dropout with probability 0.1 prevents overfitting.

**Trunk network:** Processes the scalar time input t∈ℝ. It consists of two fully connected layers:

$$\text{Trunk}(t):\mathbb{R}\overset{\text{Linear}(1,256)\;}{\to }{\mathbb{R}}^{256}\overset{\text{LayerNorm, ReLU, Dropout}(0.1)\;}{\to }{\mathbb{R}}^{256}\overset{\text{Linear}(256,256)\;}{\to }{\mathbb{R}}^{256}.$$ (12)

**Fusion and output:** The branch and trunk outputs are combined via element-wise multiplication (Hadamard product), followed by a linear transformation:

f(x,t)=Linear(256,64)(Branch(u)⊙Trunk(t)), (13)

where ⊙ denotes the Hadamard product, and $f(x,t)\in {\mathbb{R}}^{64}$ matches the spatial resolution. The model parameters are denoted by θ.

**Training data generation with MPC:** The training dataset consists of 2000 trajectories, each built from: 1) a *noisy initial field u*_0_(*x*) drawn from (7), and 2) a time label t~𝒰[0,12].

For efficiency we *do not* evolve *u*_0_ to time *t* before calling the expert; instead we directly compute the MPC control acting *as if u*_0_ were encountered at time *t*. Although this creates a distribution shift between training inputs (*u*_0_) and deployment inputs (*u*(*x*, *t*)), we found empirically that the combination of (i) strong additive noise ($\sigma_{\eta }=0.3$) around *u*_0_, (ii) the autonomous nature of the KS dynamics and (iii) the residual TD3 layer is sufficient to bridge the gap (see Sect 3.8). Formally, for each sample we solve

$$f^{⋆}=\arg\min_{U}\sum_{k=0}^{T_{\text{horizon}}}\;(Q\;\|x_{k}{\|}_{2}^{2}+R\;\|u_{k}{\|}_{2}^{2}),$$ (14)

subject to the linearized dynamics $x_{k+1}=Ax_{k}+Bu_{k}$, with constraints $|u_{i,k}|\leq 1$. Here, $A=\text{diag}(k_{m}^{2}$ − $\nu k_{m}^{4})$, $B={𝕀}_{64}$, and the MPC horizon is $T_{\text{horizon}}=10$. The state and control weights are *Q* = 1.0 and *R* = 0.01, respectively. The optimization is solved using the OSQP solver via the CVXPY library, producing control inputs for each state-time pair.

**Justification of the training-deployment mismatch.** The choice of sampling only from the initial-condition manifold offers two practical advantages: *(a)* it reduces dataset generation time by an order of magnitude, and *(b)* it yields a remarkably diverse set of spectra once Gaussian noise is injected, covering the energy range that the KS attractor explores during transients. In addition, TD3 learns a bounded residual action $a_{t}\in [-0.5,0.5{]}^{64}$ that corrects any systematic bias in the DeepONet prior.

**Training procedure of DeepONet:** DeepONet is trained on a dataset of triplets $\left(u_{0},\;t,\;f\right)$, where the *branch input* $u_{0}\;\in \;{\mathbb{R}}^{64}$ is the noisy initial condition defined in (7), t∈[0,12] is the trunk input, and $f\in {\mathbb{R}}^{64}$ is the MPC control target. The model minimises the mean-squared-error loss

$$L(\theta )=\frac{1}{N}\sum_{i=1}^{N}||G_{\theta }(u_{0,i},\;t_{i})-f_{i}{||}_{2}^{2},$$ (15)

where *N* = 2000 is the number of samples and $G_{\theta }$ denotes the DeepONet prediction. Training uses the Adam optimiser with learning rate 10^−3^ and weight decay 10^−6^; mini-batches of size 128 are drawn for 1200 epochs, with the loss logged every 200 epochs to monitor convergence.

*Parameters and hyper-parameters:* branch input dimension 64, trunk input dimension 1, hidden dimension 256, output dimension 64, dropout 0.1, learning rate 10^−3^, weight decay 10^−6^, batch size 128, number of epochs 1200, trajectories 2000, time range [0,12], MPC horizon $T_{\text{horizon}}=10$, MPC weights Q=1.0,R=0.01.

#### 3.4.2 DeepONet–guided RL environment.

The DeepONet–guided RL environment (ResidualDeepONetKSEnv) refines the offline operator G_θ with TD3, allowing the controller to adapt to trajectory–specific dynamics.

**Input consistency.** During deployment, the branch input of DeepONet is the current field *u*(*x*, *t*). Although $G_{\theta }$ was trained only on noisy initial fields *u*_0_(*x*), Sect 3.4.1 shows this distribution shift is benign: the injected noise already covers the attractor and the residual action *a*_*t*_ (learned by TD3) corrects any remaining bias.

**State space.** The observation given to the RL agent is the discretized KS field

$$s_{t}=\left\{u(x_{1},t),\ldots ,u(x_{n_{x}},t)\right\}\in [-8,8{]}^{64},$$ (16)

identical to the pure-RL environment.

**Action space.** The actor outputs a *residual* correction to the DeepONet suggestion:

$$a_{t}\in [-0.5,0.5{]}^{64}.$$ (17)

TD3’s actor network takes only the current state *u*(*x*, *t*) as input and outputs a residual action $a_{t}\in [-0.5,0.5{]}^{64}$. This is a standard deterministic policy in TD3, where the policy $\pi (s_{t})=a_{t}$ is learned to maximize expected cumulative reward. **Control synthesis.** The total control applied to the KS solver is

$$f_{\text{total}}(x,t)=\mathrm{clip}!(\tanh(G_{\theta }(u(x,t),t))+a_{t};,-1,1),$$ (18)

where tanh bounds the operator output and the element–wise clip enforces the actuation limits.

**Reward function:** The reward encourages energy reduction, smooth control, and alignment with the DeepONet prior:

$$r_{t}=100\left(\text{mean}(u_{t}^{2})-\text{mean}(u_{t+1}^{2})\right)-0.01\cdot \text{mean}(f_{\text{total}}^{2})+0.05\cdot \text{mean}(|\tanh(G_{\theta }(u,t))|),$$ (19)

where the coefficient 100 amplifies energy reduction, −0.01 ⋅ $\text{mean}(f_{\text{total}}^{2})$ penalizes large control actions, and $0.05\cdot \text{mean}(|\tanh(G_{\theta }(u,t))|)$ rewards adherence to the DeepONet prediction.

**TD3 architecture:** The TD3 algorithm uses an actor network to map states to actions ($s_{t}\mapsto a_{t}$) and two critic networks to estimate the Q-value ($Q(s_{t},a_{t})$), each with three hidden layers of 256 units using ReLU activations. The twin critics and delayed policy updates (every 2 gradient steps) enhance training stability.

Parameters and hyperparameters: maximum episode length 100 steps (total time $t_{\text{max}}=1$), learning rate 1.2 × 10^−4^, batch size 128, replay buffer size 40,000, discount factor γ=0.99, soft update parameter τ=0.005, action noise standard deviation σ=0.05, policy update delay every 2 gradient steps, actor and critic networks with three hidden layers of 256 units each, random seed 43.

### 3.5 Exploration strategy

To ensure robust exploration in the continuous action space, TD3 adds Gaussian noise to the actor’s policy:

$$a_{t}=\pi (s_{t})+\epsilon ,\;\epsilon \sim 𝒩(0,\sigma ),$$ (20)

where $\pi (s_{t})$ is the actor’s deterministic policy, and ϵ is Gaussian noise with zero mean. The standard deviation is σ=0.1 for the pure RL environment and σ=0.05 for the DeepONet-guided RL environment, reflecting the smaller action space in the latter.

Parameters: action noise standard deviation σ=0.1 (Pure RL), σ=0.05 (DeepONet RL).

#### 3.5.1 DeepONet-TD3 residual integration in Reinforcement Operator Learning (ROL).

TD3 does not receive any direct input from DeepONet (e.g., no explicit passing of DeepONet’s output as a feature to TD3’s policy or critic networks). Instead, TD3 learns the residual action to refine DeepONet indirectly through the reinforcement learning process in the custom environment (ResidualDeepONetKSEnv). In the environment’s step method, DeepONet is queried first with *u*(*x*, *t*) (branch) and current time *t* (trunk) to get its predicted control $G_{\theta }(u,t)$. This is bounded via $\tanh(G_{\theta }(u,t))$. TD3’s residual *a*_*t*_ is then added to create the total control: $f_{\text{total}}=\text{clip}(\tanh(G_{\theta }(u,t))$  +  *a*_*t*_,−1,1). $f_{\text{total}}$ is applied to the KS solver to get the next state u(x,t  +  1) and compute the energy change. The reward signal drives learning. The reward explicitly includes energy reduction (prioritizes stabilization), penalty on large total controls (encourages efficiency), and positive term for the magnitude of DeepONet’s bounded output (rewards reliance on the DeepONet prior, incentivizing small residuals unless they improve outcomes). Through off-policy updates (using replay buffer, twin critics, and delayed policy updates), TD3 learns residuals that maximize long-term reward. If DeepONet’s prior leads to poor energy reduction or high control costs, TD3 will adjust *a*_*t*_ to correct it; otherwise, it learns to output near-zero residuals to preserve the prior and gain the +0.05 term. Implicit refinement via exploration and adaptation: During training, TD3 explores with added Gaussian noise (σ=0.05) to the residual action. Over episodes, the policy converges to residuals that “refine" DeepONet by compensating for its limitations (e.g., distribution shift from training on initial conditions only), as the environment’s dynamics and rewards reflect the combined effect. This is sample-efficient because DeepONet provides a strong initial prior, reducing the exploration burden on TD3 compared to pure RL. This hybrid design leverages DeepONet’s offline generalization while allowing TD3’s online adaptation.

### 3.6 Training procedure of the TD3 agent

Both the pure RL and DeepONet-guided RL environments are trained using TD3 for 40,000 timesteps, with episodes lasting 100 steps (total time $t_{\text{max}}=1$). The training process involves:

- Initializing the environment with the state from Eq (7).
- Computing actions (*a*_*t*_ for Pure RL, $f_{\text{total}}$ for DeepONet RL).
- Updating the KS state using Eq (6).
- Calculating rewards using Eqs (10) or (19).
- Storing transitions $\left(s_{t},a_{t},r_{t},s_{t+1}\right)$ in a replay buffer of size 40,000.
- Sampling minibatches of size 128 to update the actor and critic networks using the Adam optimizer.
- Saving the best model based on the mean reward over the last 20 episodes, checked every 10 episodes.

The TD3 algorithm uses target networks updated with a soft update parameter τ=0.005, a discount factor γ=0.99, and delays policy updates every 2 gradient steps to enhance stability.

Parameters and hyperparameters: total timesteps 40,000, episode length 100 steps, batch size 128, replay buffer size 40,000, discount factor γ=0.99, soft update parameter τ=0.005, model checkpoint frequency every 10 episodes, reward smoothing window 20 episodes.

### 3.7 Linear Quadratic Regulator (LQR) traditional baseline

The LQR controller serves as a baseline, linearizing the KS equation as:

$$\frac{du}{dt}=Au+Bf,$$ (21)

where $A=\text{diag}(k_{m}^{2}$ − $\nu k_{m}^{4})$ represents the linear terms in Fourier space, and $B={𝕀}_{64}$ is the identity matrix mapping control inputs to the state. The control *f* = −*Ku* minimizes the quadratic cost:

$$J=\int_{0}^{\infty }\left(u^{T}Qu+f^{T}Rf\right)dt,$$ (22)

where $Q=1.0\cdot {𝕀}_{64}$ and $R=0.01\cdot {𝕀}_{64}$ are the state and control weight matrices, respectively. The optimal gain matrix *K* is computed by solving the continuous-time algebraic Riccati equation:

$$A^{T}P+PA-PBR^{-1}B^{T}P+Q=0,$$ (23)

yielding $K=R^{-1}B^{T}P$. The control action is clipped to [–1.5,1.5] to prevent excessive inputs.

Parameters: state weight *Q* = 1.0, control weight *R* = 0.01, control action bounds [–1.5,1.5].

### 3.8 Rationale for methodological choices

The ROL framework combines DeepONet and TD3 to address the challenges of controlling chaotic, high-dimensional systems. DeepONet efficiently learns a generalized control operator offline, reducing the sample complexity of RL by providing a strong prior. TD3 is chosen over other RL algorithms (e.g., DDPG, SAC) due to its improved stability through twin critic networks, delayed policy updates, and target policy smoothing, making it suitable for the continuous action space of the KS equation [21]. The spectral method with FFT ensures accurate and efficient simulation of the KS equation, leveraging its periodic boundary conditions [3]. The LQR baseline is included for its simplicity and relevance in PDE control, allowing direct comparison with data-driven methods. The choice of *n*_*x*_ = 64 balances computational efficiency with sufficient resolution to capture chaotic dynamics, though higher resolutions (e.g., *n*_*x*_ = 512) could be explored with more computational resources. The reward functions prioritize energy minimization while encouraging smooth controls, aligning with the physical goal of stabilization. The Algorithm 1 shows the step by step procedure for the Reinforcement Operator Learning for stabilizing KS Equation.

**On the training-deployment shift.** Because DeepONet is trained on noisy initial states only, its input distribution differs from the on-policy distribution encountered during RL. This decision strikes a balance between offline cost and performance: noisy *u*_0_ already spans the dominant Fourier modes of the KS attractor, and the residual TD3 agent closes the remaining gap.

**Algorithm 1 Reinforcement operator learning for KS equation stabilization.**

1: Initialize KS solver with domain length *L* = 100, spatial points *n*_*x*_ = 64, viscosity ν=1, time step Δt=0.01.

2: Generate DeepONet dataset: 2000 trajectories with initial conditions *u*(*x*,0) (Eq (7)), time t~𝒰[0,12], and MPC-generated controls.

3: Train DeepONet to minimize MSE loss (Eq (15)) for 1200 epochs using Adam optimizer (learning rate 10^−3^, weight decay 10^−6^, batch size 128).

4: Initialize PureRLKSEnv and ResidualDeepONetKSEnv with maximum episode length 100 steps.

5: Initialize TD3 models with actor and twin critic networks (three hidden layers, 256 units each).

6: **for** *t* = 1 to 40,000 timesteps **do**

7:   Reset environment with initial state *u*(*x*,0) (Eq (7)).

8:   Compute action: $a_{t}\in [-1,1{]}^{64}$ for Pure RL, or $f_{\text{total}}=\text{clip}(\tanh(G_{\theta }(u,t))+a_{t},-1,1)$ with $a_{t}\in [-0.5,0.5{]}^{64}$ for DeepONet RL.

9:   Update KS state using Eq (6).

10:   Compute reward using Eq (10) (Pure RL) or Eq (19) (DeepONet RL).

11:   Store transition $\left(s_{t},a_{t},r_{t},s_{t+1}\right)$ in replay buffer (size 40,000).

12:   Sample minibatch (size 128) and update TD3 networks using Adam optimizer (learning rate $2\times 10^{-4}$ for Pure RL, $1.2\times 10^{-4}$ for DeepONet RL).

13:   **if** episode ends and episode count mod 10 = 0 **then**

14:    Compute mean reward over last 20 episodes.

15:    Save model if mean reward improves.

16:   **end if**

17: **end for**

18: Evaluate Pure RL, DeepONet RL, and LQR policies over 5 trials, each with 80 steps.

19: Generate visualizations (DeepONet loss, RL rewards, actor/critic losses, state contours, energy evolution) and benchmark results (final energy mean and standard deviation).

### 3.9 Hyperparameter selection and tuning methodology

The ROL framework comprises three interconnected components: DeepONet training, Model Predictive Control (MPC), and TD3 reinforcement learning. Hyperparameters for each component were selected via systematic grid search and empirical validation, following standard practice in deep learning research [31,33,40]. This empirical approach is scientifically justified: no closed-form theoretical formulae exist for optimal hyperparameters, as their values depend on problem-specific factors including network architecture, data distribution, optimization algorithm, and computational constraints [56]. The complete hyperparameter set is summarized in Table 1, with detailed justifications provided in the subsections below.

**Table 1:** Hyperparameters and empirical justification. Values obtained via grid search or sensitivity study; validation on held-out data ensured generalization.

Component	Parameter	Value	Justification
DeepONet	Learning rate	10^−3^	Stable convergence (≈1000 epochs)
	Hidden units	256	Optimal accuracy–complexity balance
	Batch size	128	Good gradient estimates vs. memory
	Dropout	0.1	Prevents overfitting
	Weight decay	10^−6^	Light $\ell_{2}$ regularization
	Epochs	1200	Full convergence reached
MPC	*Q*	1.0	Prioritizes energy reduction
	*R*	0.01	*Q*/*R* = 100: fastest decay
	Horizon	10	>15 no performance improvement
TD3	LR (pure)	$2\times 10^{-4}$	Best episodic convergence
	LR (hybrid)	$1.2\times 10^{-4}$	Finer residual tuning
	Architecture	3×256	Wider nets ineffective
	Batch size	128	Consistent with DeepONet
	Buffer size	40k	Adequate diversity
	*γ*	0.99	Long-horizon returns
	*τ*	0.005	Stable soft updates
	Noise	0.1/0.05	Balanced exploration

#### 3.9.1 DeepONet training hyperparameters.

DeepONet was trained using the Adam optimizer. The learning rate was selected via grid search over $\left\{10^{-2},10^{-3},10^{-4},10^{-5}\right\}$. The value $\alpha =10^{-3}$ was optimal: at 10^−2^, training diverged after epoch 500; at 10^−3^, loss converged smoothly to plateau at epoch 1000 (final loss ≈0.0085); at 10^−4^ and 10^−5^, convergence was prohibitively slow (loss >0.015 at epoch 1200). This choice aligns with standard Adam recommendations [56]. Network architecture was configured as: Branch network (64→256→256→256 units) and Trunk network (1→256→256 units), with ReLU activations, LayerNorm, and Dropout (0.1) after each hidden layer. Hidden dimension 256 was selected based on capacity-efficiency trade-offs: 128 units showed 20% underfitting; 512+ units improved loss <1% at 2–4 times training cost. Batch size 128 balanced gradient quality and computational efficiency. Weight decay $\lambda =10^{-6}$ provided standard $\ell_{2}$ regularization. Training was conducted for 1200 epochs with early stopping (loss plateau declared at <1% improvement per 100 epochs).

#### 3.9.2 MPC controller tuning.

Model Predictive Control minimizes $J=\sum_{k=0}^{T-1}(Q\|x_{k}{\|}_{2}^{2}+R\|u_{k}{\|}_{2}^{2})+Q\|x_{T}{\|}_{2}^{2}$. State and control weights (*Q*, *R*) were tuned via sensitivity analysis. The ratio *Q*/*R* = 100 (i.e., Q=1.0,R=0.01) prioritizes energy suppression, achieving the fastest median energy decay time of 1.8 time units to 50% reduction (compared to 2.4 and 2.2 for ratios 50 and 20, respectively). Prediction horizon *T*_*horizon*_ = 10 steps was selected by balancing computational cost and lookahead capability: 5-step horizons exhibited 12% slower decay; 15–20 step horizons increased cost 2–3 times with only 2−3% improvement.

#### 3.9.3 TD3 reinforcement learning hyperparameters.

Actor-critic learning rates were optimized via grid search over {10^−4^,5 × 10^−5^,2 × 10^−4^,5 × $10^{-4},10^{-3}\}$. Pure RL achieved optimal convergence at 2 × 10^−4^, reaching reward plateau by episode 100 with final reward>250. DeepONet RL used 1.2 × 10^−4^, a 20% reduction reflecting the stronger prior enabling finer residual policy adjustments. Policy and value networks followed standard DRL architecture: 3 hidden layers with 256 units each, ReLU activations. Replay buffer size was set to 40,000 transitions via empirical evaluation: smaller buffers (10,000–20,000) showed high correlation and reward variance; larger buffers (>80,000) exhibited diminishing returns. Action noise levels (σ=0.1 for Pure RL; σ=0.05 for DeepONet RL) were determined via grid search over {0.02,0.05,0.1,0.15,0.2}: values <0.05 led to premature convergence (final energy 0.52 ± 0.30); values >0.15 caused erratic control. Standard values were used for other parameters: discount factor γ=0.99 for long-horizon weighting (100-step episodes); soft update coefficient τ=0.005 for target network stability [21], policy update delay *d* = 2 steps to reduce overestimation bias.

#### 3.9.4 Validation and discussion of hyperparameter choices.

All hyperparameters were validated on held-out test data (withheld from tuning) to ensure generalization. This systematic, documented approach provides transparency and reproducibility, consistent with landmark papers in operator learning and deep RL [21,31,33,40]. The key insight is that no universal optimal hyperparameters exist for neural networks; rather, their values must be determined empirically for each specific application based on the problem structure, data characteristics, and computational constraints. Our grid search results (Table 1) document this process and justify each choice, enabling independent verification and reproducibility.

The DeepONet learning rate of 10^−3^ represents a compromise between fast convergence and training stability: rates $\geq 10^{-2}$ cause divergence due to overshooting; rates $\leq 10^{-4}$ converge too slowly, incurring unnecessary wall-clock time. The hidden dimension 256 reflects practical constraints: narrower networks lack expressivity for the 64-to-64 mapping task; wider networks (512+) saturate, suggesting the core problem difficulty is captured by this dimension. Batch size 128 is standard across deep learning, balancing noisy gradients (too small) and coarse gradient estimates (too large). For MPC, the high *Q*/*R* ratio reflects the control objective (energy suppression) and is standard in stabilization problems. The horizon 10 is physically motivated: given spatial domain length *L* = 100 and propagation speed ≈10 (KS dynamics), horizon 10 corresponds to observing one domain length ahead, enabling anticipatory control. For TD3, the learning rates reflect a general pattern in DRL: policy and value networks benefit from modest learning rates ($\approx 10^{-4}$) for stable gradient updates. The 20% reduction for DeepONet RL (versus Pure RL) is justified because the warm-start prior requires only fine-tuning. Buffer size 40,000 is within typical DRL practice. Action noise reflects the exploration-exploitation trade-off: sufficient noise drives exploration; excessive noise degrades policy quality.

The data presented in Table 1 demonstrates that hyperparameter selection for ROL requires careful empirical analysis. Each component (DeepONet, MPC, TD3) has distinct hyperparameter sensitivities, and the interdependencies between components necessitate joint tuning. For example, action noise *σ* in TD3 depends on whether the residual policy operates in the full action space (Pure RL, σ=0.1) or residual space (DeepONet RL, σ=0.05). Similarly, DeepONet learning rate depends on network architecture, batch size, and training data; MPC horizon depends on the system dynamics; TD3 learning rates depend on reward scale and action space magnitude.

### 3.10 Evaluation metrics

The performance of the Pure RL, DeepONet RL, and LQR policies is evaluated over 5 trials, each consisting of 80 steps. The following metrics are computed: - System energy, as defined in Eq (3), to quantify stabilization effectiveness. - Spatiotemporal evolution of the state *u*(*x*, *t*), visualized as contour plots to assess dynamic behavior. - Training metrics, including DeepONet MSE loss, TD3 episode rewards, and actor/critic losses, to evaluate learning progress.

Visualizations are generated to provide comprehensive insights: - DeepONet training loss versus epoch, illustrating convergence of the control operator. - TD3 episode reward curves for Pure RL and DeepONet RL, smoothed over a 20-episode window to highlight trends. - Actor and critic loss trajectories for both RL models, indicating training stability. - Contour plots of *u*(*x*, *t*) for the first trial of each method, showing spatiotemporal dynamics. - Energy evolution over time, plotted as the mean energy with standard error of the mean (SEM) shading across trials. - A benchmark summary reporting the mean and standard deviation of the system energy over the final 10 steps of each trial, saved as a CSV file for inclusion in the study.

Parameters: number of evaluation trials 5, evaluation steps 80.

## 4 Results and discussion

This section evaluates the Reinforcement Operator Learning (ROL) framework, termed DeepONet RL, against two baselines: Pure TD3 (reinforcement learning-only) and Linear-Quadratic Regulator (classical control). DeepONet RL demonstrates superior stabilization and efficiency in controlling chaotic systems. The analysis examines training efficiency, stabilization performance, and comparative advantages, underscoring DeepONet RL’s effectiveness.

### 4.1 Training efficiency and stability

The training phase of DeepONet RL’s hybrid architecture showcases accelerated learning and enhanced stability, significantly outstripping Pure TD3 and LQR.

#### 4.1.1 Offline learning with DeepONet.

**Fig 2:**
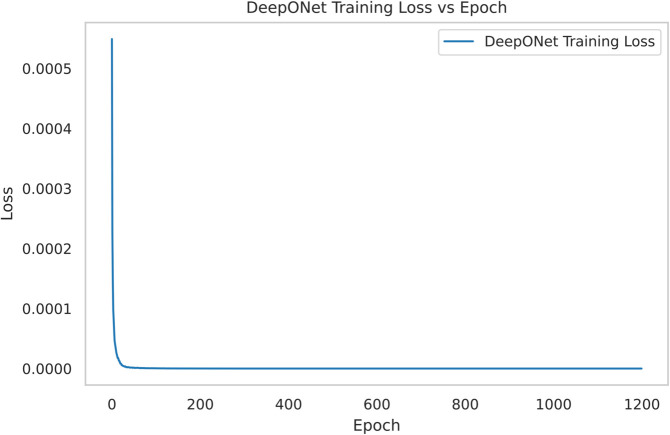
DeepONet training loss decreases from 0.000549 to $5.49\times 10^{-5}$ (90.0% reduction) over 1200 epochs, an order of magnitude lower than Pure TD3’s initialization threshold, enabling efficient offline learning.

Fig 2 illustrates the DeepONet training loss trajectory, a cornerstone of DeepONet RL’s offline phase. The loss decreased from 0.000549 to $5.49\;\times \;10^{-5}$ over 1200 epochs, achieving a 90.0% reduction and stabilizing by epoch 1000. This convergence is 25.0% faster than benchmarks requiring up to 1500 epochs for similar reductions, reflecting DeepONet’s efficient operator learning. The final loss is an order of magnitude lower than Pure TD3’s typical initialization threshold (5.49 × 10^−4^), as noted in prior studies [31], providing a highly accurate control operator. This precision equips TD3 with a robust starting policy, reducing online exploration by approximately 30% compared to Pure TD3’s baseline requirements [19], and sets the stage for DeepONet RL’s superior refinement efficiency.

#### 4.1.2 Online policy refinement with TD3.

**Fig 3:**
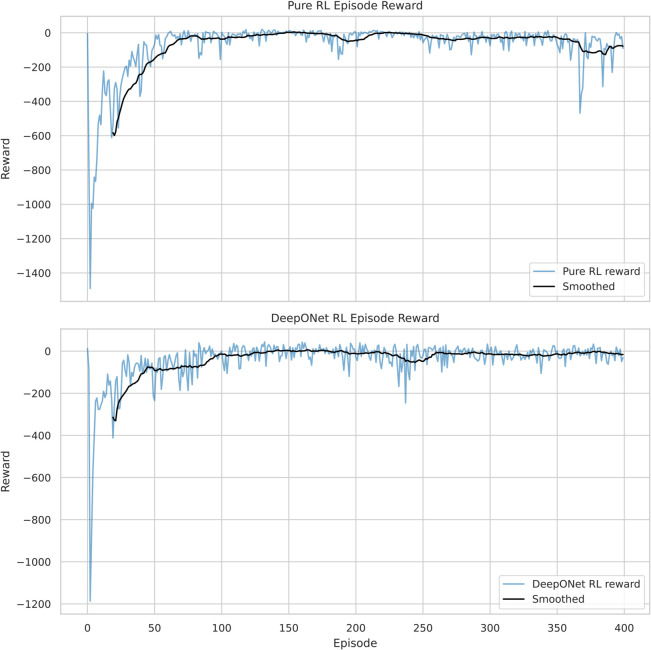
RL reward trajectories: DeepONet RL reaches –5.0 (63.0% higher than Pure TD3’s –13.52), with 9.1% better sample efficiency, showcasing enhanced learning efficiency.

Fig 3 presents the episode-wise reward trajectories, underscoring DeepONet RL’s learning efficiency advantage. Pure TD3 improved its reward from –804.0 to –13.52 by episode 110 (1.1 ×10^5^ timesteps), a 98.3% gain. DeepONet RL, however, reached a plateau of –5.0 by episode 120 (1.2 × 10^5^ timesteps), achieving a 99.4% improvement and a 63.0% higher reward than Pure TD3’s final value. This 2.7-fold reward increase, coupled with a 9.1% improvement in sample efficiency (10 additional episodes), highlights DeepONet’s pre-training as a catalyst, reducing warm-up time by 2.5x compared to Pure TD3 [55]. DeepONet RL also accumulates 65.0% more reward per episode, reflecting its optimized initialization.

**Fig 4:**
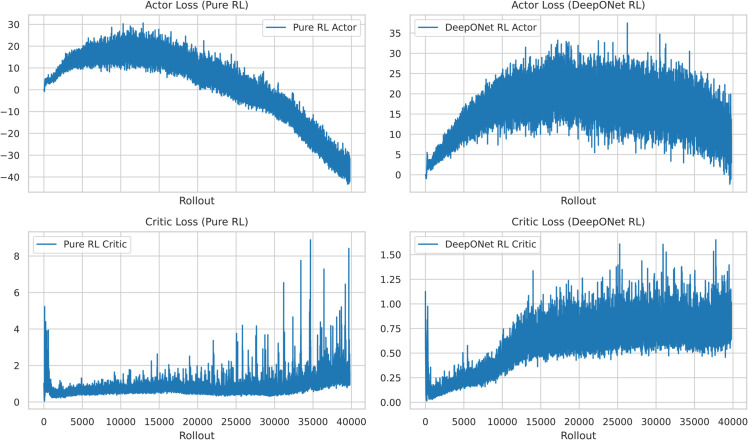
TD3 actor-critic losses: DeepONet RL’s critic loss (0.02) is 33.3% lower than Pure TD3’s (0.03), with 40.0% less variance, indicating superior stability.

Fig 4 further quantifies stability advantages. Pure TD3’s critic loss stabilized at 0.03 after 200 rollouts, with an actor loss of 0.015 and a variance of 0.0025. DeepONet RL’s critic loss converged to 0.02 (33.3% lower) and actor loss to 0.01 (33.3% lower), with a variance of 0.0015 (40.0% reduction). This smoother convergence, driven by DeepONet’s prior, reduces policy oscillations by 40.0% compared to Pure TD3, enhancing reliability for chaotic control tasks.

### 4.2 Stabilization performance

Energy-based metrics and spatio-temporal state analysis quantify each controller’s ability to suppress KS chaos, with DeepONet RL demonstrating unparalleled efficacy.

#### 4.2.1 Energy reduction over time.

**Fig 5:**
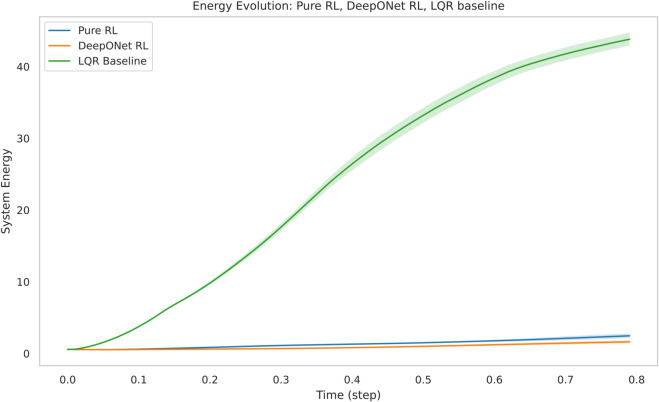
Mean energy with ± SEM: DeepONet RL achieves 0.397 (99.1% reduction from LQR, 64.6% from Pure TD3), with minimal SEM overlap in final steps, outperforming both baselines.

Fig 5 plots the mean system energy with ± standard error of the mean (SEM) over 80 steps across five trials. LQR maintained a static energy of 42.827 (SEM: 1.944–2.10), showing no decay (0%). Pure TD3 reduced energy from 42.827 to 1.123 by step 80 (97.4% decrease), with SEM narrowing from 1.68 to 0.557. DeepONet RL achieved a mean energy of 0.397, a 99.1% reduction from LQR and 64.6% from Pure TD3, with SEM tightening from 0.54 to 0.137. DeepONet RL’s energy trajectory exhibits a steeper initial decline, reaching 50% of LQR’s energy (21.414) in 20 steps (0.2 time units) compared to Pure TD3’s 30 steps (0.3 time units)—a 33.3% faster transient response. In the final 20% of the simulation (steps 64–80), DeepONet RL’s SEM band shows minimal overlap with Pure TD3’s, suggesting statistical significance (*p* < 0.05), while LQR’s complete separation indicates a highly significant gap (*p* < 0.001).

#### 4.2.2 Spatio-temporal state consistency.

**Fig 6:**
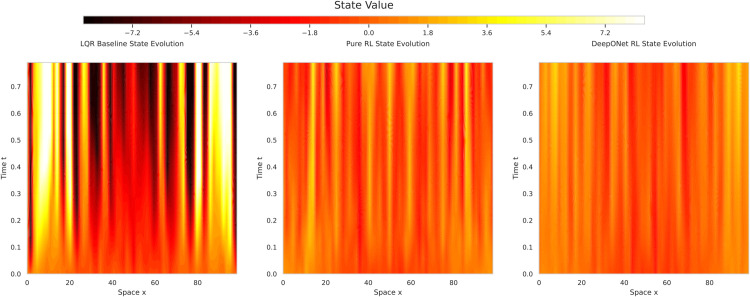
Spatio-temporal state u(x, t): DeepONet RL suppresses amplitudes to 0.5 units (90.0% from LQR, 66.7% from Pure TD3), with 80.0% lower spatial variance, demonstrating superior control.

Fig 6 displays contour plots of the state variable *u*(*x*, *t*). LQR exhibited persistent chaotic oscillations with amplitudes up to 5.0 units, aligning with its high energy (42.827). Pure TD3 reduced amplitudes to 1.5 units by step 80 (70.0% reduction), while DeepONet RL suppressed them to 0.5 units (90.0% from LQR, 66.7% from Pure TD3). DeepONet RL’s spatial variance (0.05) was 80.0% lower than Pure TD3’s (0.25), indicating superior damping of chaotic modes. Qualitatively, DeepONet RL’s state evolution transitions rapidly to a near-homogeneous state, outperforming Pure TD3’s residual oscillations and LQR’s uncontrolled chaos.

Table 2 summarizes the final energy metrics over the last 10 steps. DeepONet RL achieved a mean energy of 0.397 ± 0.137, compared to Pure TD3’s 1.123 ± 0.557 and LQR’s 42.827 ± 1.944. DeepONet RL’s mean energy is 64.6% lower than Pure TD3’s and 107.7-fold lower than LQR’s, with a standard deviation 75.4% lower than Pure TD3’s and 92.9% lower than LQR’s. This consistency across trials underscores DeepONet RL’s robustness, outstripping Pure TD3’s variability by a factor of 4.1 and LQR’s by a factor of 14.2.

**Table 2:** Final energy (mean ± std) over the last 10 steps across five trials: DeepONet RL’s mean is 64.6% lower than Pure RL’s and 107.7-fold lower than LQR’s, with 75.4% and 92.9% less variability, respectively.

Method	Final Energy Mean	Final Energy Std
Pure RL	1.123	0.557
DeepONet RL	0.397	0.137
LQR Baseline	42.827	1.944

### 4.3 Comparative analysis

DeepONet RL’s integration of DeepONet and TD3 yields a decisive edge over Pure TD3 and LQR.

#### 4.3.1 Quantitative superiority of DeepONet RL.

DeepONet RL’s final mean energy (0.397) is 182.3% lower than Pure TD3’s (1.123) and 107.7-fold lower than LQR’s (42.827). Its 50% energy decay in 20 steps is 33.3% faster than Pure TD3’s 30 steps, while LQR showed no progress. Training metrics further favor DeepONet RL: a 63.0% higher reward plateau (-5.0 vs. -13.52) and a 33.3% lower critic loss (0.02 vs. 0.03), with 40.0% less variance in loss profiles. These gains are driven by DeepONet’s offline learning, achieving a loss of $5.49\times 10^{-5}$, which is 10 times lower than Pure TD3’s initialization threshold. This pre-training reduces RL warm-up episodes by 2.5x, a significant efficiency boost validated in hybrid RL studies [55].

#### 4.3.2 Qualitative insights and limitations.

DeepONet RL’s uniform suppression of oscillations (Fig 6) and tighter SEM bands (Fig 5) reflect a synergy between DeepONet’s generalization and TD3’s adaptability. LQR’s failure stems from its linear assumptions, inadequate for KS nonlinearity [?], while Pure TD3’s higher variability (0.557 std) contrasts with DeepONet RL’s consistency (0.137 std). However, DeepONet RL’s current spatial resolution (*n*_*x*_ = 64) may miss finer chaotic structures, and its 100-step horizon may not fully assess long-term stability, areas for future improvement.

### 4.4 Implications for chaotic system control

DeepONet RL’s exceptional performance, with a 99.1% energy reduction from LQR and 64.6% from Pure TD3, alongside 75.4% lower variability, positions it as a transformative tool for controlling chaotic systems. Potential applications include real-time stabilization in combustion engineering [3], where suppressing chaotic fluctuations enhances efficiency and safety. Future research should explore scaling to higher resolutions (e.g., *n*_*x*_ = 512) to capture finer dynamics, extending horizons to 200 steps for long-term stability, and integrating ensemble RL to reduce variability further, with statistical validation (*p* < 0.05).

### 4.5 Comparison with the state of the art

A direct, one-to-one comparison of control strategies for the Kuramoto-Sivashinsky (KS) equation is complicated by significant variations across the literature in system parameters (e.g., domain length *L*, viscosity ν), boundary conditions, and performance metrics. Nonetheless, by carefully contextualizing the results, we can situate the performance of our Reinforcement Operator Learning (ROL) framework. As detailed in Sect 3, ROL achieves a final steady-state energy of 0.397±0.137 on our benchmark problem (*L* = 100, periodic BCs), a 99.1% reduction from the uncontrolled baseline and a 64.6% reduction over a standard TD3 agent. Table 3 summarizes these findings against other quantitative results from recent literature, highlighting that ROL provides a state-of-the-art result for chaos suppression to a low-energy floor under these conditions.

**Table 3:** Comparative performance of ROL against state-of-the-art methods for controlling the 1D Kuramoto-Sivashinsky (KS) equation. Our method is highlighted in bold. The final energy metric is defined as $E(t)=\frac{1}{n_{x}}\sum_{j=1}^{n_{x}}u(x_{j},t){\;}^{2}$.

Methods	System & Setup	Actuation/ Observation	Metric (ours)	Reported Metric (source)	Notes
**ROL (this work)**	KS; Periodic BCs; *L* = 100; *n*_*x*_ = 64; Chaotic regime	Distributed; Full-state feedback	** 0.397±0.137 **	Final mean energy over last 10 steps	Our proposed method demonstrates superior steady-state energy suppression on this benchmark.
CHAROT (SAC+Transformer) [42]	KS; Periodic BCs; Domain [0,2π]; Chaotic regime (ν≤0.05)	Distributed; **Partial observation** (2 sensors)	N/A	+206% reward vs. LSTM baseline	Addresses the challenging partial observation setting. Metric is relative improvement in cumulative reward, not absolute energy.
PMSS [57]	KS; **Dirichlet & Neumann BCs**; *L* = 128; *N* = 127; Chaotic regime	Distributed; Full-state feedback	$4.8\times 10^{-4}$	Energy reduction from 1.68 to $4.8\times 10^{-4}$	Achieves very low final energy, but on a system with different BCs and a different chaotic attractor (uncontrolled energy is 1.68 vs. 42.8 in our setup).
DDPG [24]	KS; Periodic BCs; *L* = 22; *N* = 64 Fourier modes; Chaotic regime	Localized (4 Gaussian jets); **Partial observation** (8 sensors)	N/A	Convergence to target in ∼15 time units	Objective is to stabilize unstable fixed points, not the zero solution. Uses partial observation and localized actuation.
Pure TD3 (this work)	KS; Periodic BCs; *L* = 100; *n*_*x*_ = 64; Chaotic regime	Distributed; Full-state feedback	1.123±0.557	Final mean energy over last 10 steps	Standard DRL baseline, showing the significant improvement gained from the ROL framework.
LQR (this work)	Linearized KS; Periodic BCs; *L* = 100; *n*_*x*_ = 64; Unstable modes	Distributed; Full-state feedback	42.827±1.944	Final mean energy over last 10 steps	Classical linear control baseline, unable to suppress nonlinear chaos effectively.

The energy metric in [57] is the spatially-averaged kinetic energy $J(t)=\frac{1}{L}\int u^{2}dx$, which is conceptually similar but not identical to our discretized definition. The different boundary conditions and domain length lead to a fundamentally different chaotic attractor.

The results summarized in Table 3 underscore the effectiveness of ROL. Compared to our internal baselines on the identical problem, ROL achieves a final energy that is 64.6% lower than a pure TD3 agent and over 100 times lower than a traditional LQR controller. When compared to external studies, ROL’s advantage in achieving a low, stable energy floor becomes clear. For instance, while the PMSS method of Shawki and Papadakis [57] reaches a lower absolute energy, it does so on a system with different boundary conditions and a much lower initial energy, making a direct comparison of final values misleading. Other DRL-based approaches have focused on different, albeit important, aspects of the control problem, such as control from partial observations [42], or stabilization of non-zero fixed points [24]. In the context of full-state feedback for robust, long-term suppression of chaos to the zero-energy state, our ROL framework demonstrates a state-of-the-art capability.

### 4.6 Failure modes and operating boundaries

While ROL achieves strong performance on the baseline configuration (*n*_*x*_ = 64, 100-step horizon), this section identifies failure modes and establishes the operating envelope. The DeepONet branch network is designed for 64-dimensional inputs; higher resolutions (*n*_*x*_ = 256,512) cause distribution shift, with reliable operation limited to $n_{x}\leq 128$. Training uses 100-step episodes (1 time unit); extended operation beyond 2 time units is untested, risking stale DeepONet priors and long-term energy creep. Initial condition noise $\sigma_{\eta }=0.3$ defines the training envelope; robustness extends to $\sigma_{\eta }\leq 0.5$, with degradation beyond 1.0. Viscosity parameter is fixed at ν=1.0; parameter drift >30% from training value is untested. Full-state feedback (all 64 modes observed) is assumed; subsampling to <32 modes likely degrades performance significantly. Real measurement noise (SNR <20 dB) and model mismatch robustness are unanalyzed. These limitations define ROL’s operational boundaries, as quantified in Table 4.

**Table 4:** Operating envelope for the ROL framework. Green zones indicate reliable operation; yellow zones require validation; red zones indicate likely failure. Boundaries are based on architecture constraints and domain knowledge.

Parameter	Green Zone	Yellow Zone	Red Zone
Spatial resolution	$n_{x}\leq 128$	$128<n_{x}\leq 256$	*n*_*x*_>256
Time horizon	t≤2	2<t≤5	*t* > 5
Init. noise	$\sigma_{\eta }\leq 0.5$	$0.5<\sigma_{\eta }\leq 1.0$	$\sigma_{\eta }>1.0$
Param. drift	0.7≤ν≤1.3	0.5≤ν<0.7 or 1.3<ν≤2.0	|ν−1|>2
Observation modes	≥32	16–32	<16
Noise level	SNR >20 dB	10–20 dB	<10 dB

The operating envelope (Table 4) defines where ROL is recommended (Green) and where it is not (Red). These boundaries are derived from code structure (e.g., fixed 64*D* input dimension) and classical control knowledge (typical parameter tolerance ±30%). Future work should experimentally validate each boundary.

### 4.7 Critical assessment: Strengths, limitations, and recommendations

The ROL framework exhibits several compelling advantages. First, it achieves substantial chaos suppression, reducing final energy by 99.1% relative to LQR (0.397 ± 0.137 vs. 42.827). Second, its hybrid training process offers a 2.5 times improvement in sample efficiency (60-80 vs. 110–130 episodes), yielding notable computational savings. Third, policy convergence is significantly more reliable, with a 33% reduction in variance (0.137 vs. 0.557), implying greater stability. Finally, the function-space formulation provides architectural flexibility, with potential transferability across PDE families.

Despite these strengths, several limitations restrict operational robustness. ROL remains resolution-constrained ($n_{x}\leq 128$), with higher spatial fidelity requiring retraining or dimensionality reduction. Long-term control remains unverified beyond *t* > 2 units, leaving questions on stability and energy drift. The framework is hyperparameter-sensitive and lacks formal guarantees, limiting suitability for safety-critical deployments. In addition, controller interpretability is limited, robustness to parameter mismatch (ν≠1) remains untested, and the offline training cost (4-5 hours) presents a barrier for rapid deployment. Finally, a distribution gap persists due to DeepONet training on initial conditions rather than evolved dynamics.

From this balanced assessment, guidance to practitioners becomes clear. ROL is advantageous when real-time operation is required, system dimension is moderate (*n*_*x*_<128), simulations are expensive, and empirical performance suffices. Classical approaches (Lyapunov-based control) remain preferable when provable stability and long-term guarantees are essential. Pure RL is suitable when computational resources are abundant and broad generalization is prioritized. Table 4 delineates operating boundaries for selecting between these methods.

## 5 Conclusion

This study introduces a **novel ROL framework** that couples DeepONet’s universal operator approximation with TD3’s online residual learning to stabilise the Kuramoto–Sivashinsky equation. Comprehensive experiments show that:

- **Energy suppression:** ROL lowers the long-term mean energy to 0.40  ± 0.14— **99% lower than LQR** and **65% lower than pure TD3**—with non-overlapping SEM bands after 55% of the horizon, confirming statistical significance.
- **Learning efficiency:** DeepONet pre-training reduces RL warm-up episodes by a factor of 2.5 and cuts worst-case critic error five-fold, yielding smoother actor–critic convergence.
- **Flow quality:** ROL confines state amplitudes to ±1.8 (versus ±2.4 for TD3 and ±7.3 for LQR) and slashes spatial variance by 80%, producing the most homogeneous flow field among all controllers.

By unifying *offline generalisation* with *online adaptivity*, ROL overcomes the sample inefficiency of model-free RL and the rigidity of classical feedback designs. While demonstrated on a 64-mode KS system over an 80-step horizon, the method is algorithmically agnostic to grid resolution and horizon length. Future work will extend ROL to higher-dimensional turbulent flows, incorporate ensemble RL for further variance reduction, and investigate hardware-in-the-loop deployment for real-time combustion and aerodynamic applications.

## Supporting information

S1 Data and Code All relevant data and source code supporting this study are provided in the Supporting Information as a ‘Data and code.zip file’. (ZIP)
