# Supplementary material for: Reinforcement Operator Learning (ROL): A hybrid DeepONet-guided reinforcement learning framework for stabilizing the Kuramoto–Sivashinsky equation
Source: PLoS One. 2026 Jan 30;21(1):e0341161. doi: 10.1371/journal.pone.0341161 (PMC12858074; doi:10.1371/journal.pone.0341161)
Supplement: Attachment — Submitted filename: Review-Response.docx [file pone.0341161.s002.docx]

**Responses to reviewer’s comments**

**Title:** Reinforcement Operator Learning (ROL): A Hybrid DeepONet-Guided Reinforcement Learning Framework for Stabilizing the Kuramoto–Sivashinsky Equation.

**Ref:** PONE-D-25-52573

**ACADEMIC EDITOR:**

**Q1:** The state of the art should be improved, including references from 2025.

**Response:** We have substantially enhanced the Introduction section with new references from 2024-2025.

**Q2:** Present more clearly how the hyperparameter tuning was performed.

**Response:** We have added a comprehensive new subsection (Section 3.9) entitled "Hyperparameter Selection and Tuning Methodology" that systematically documents the grid search process for all hyperparameters across three components (DeepONet, MPC, TD3).

Detailed explanation includes:

- DeepONet: Learning rate ${10}^{-3}$ (grid search over {${10}^{-2}, {10}^{-3}, {10}^{-4}, {10}^{-5}\}$), hidden dimension 256, batch size 128, dropout 0.1, training 1200 epochs.
- MPC: Weights Q=1.0, R=0.01 (ratio 100), horizon 10 steps.
- TD3: Learning rates ($2\times{10}^{-4}$ pure, $1.2\times{10}^{-4}$DeepONet), action noise (0.1 pure, 0.05 DeepONet), replay buffer 40,000.

**Q3:** Include failure or instability cases, exploring where the ROL performs worse.

**Response:** We have added a new subsection (Section 4.6) "Failure Modes and Operating Boundaries" that systematically explores six failure scenarios:

1. Resolution dependence: Reliable operation limited to $n_{x}\leq128$; higher resolutions cause distribution shift.
2. Temporal horizon constraints: Untested beyond 2-time units; long-term stability unknown.
3. Noise and parameter sensitivity: Robustness to $\sigma_{\eta}\leq0.5$; parameter drift >30% untested.
4. Observation constraints: Performance degrades below 32 modes (50% coverage).
5. Measurement noise: Real noise (SNR < 20 dB) causes degradation.
6. Model mismatch robustness: Fixed parameters; unanalyzed robustness.

**Q4:** Include a more balanced discussion, highlighting both strengths and limitations.

**Response:** We have added a comprehensive new subsection (Section 4.7) "Critical Assessment: Strengths, Limitations, and Recommendations" providing balanced assessment.

Strengths covered:

1. Energy suppression: 99.1% reduction vs. LQR (0.397 vs. 42.827).
2. Sample efficiency: 2.5 times faster convergence (60-80 vs. 110-130 episodes).
3. Policy stability: 33% lower variance (0.137 vs. 0.557).
4. Architectural flexibility: Function-space learning, potential generalization.

Limitations covered:

1. Resolution dependency ($n_{x}\leq128$).
2. Temporal horizon ($t\leq2$ units).
3. Hyperparameter sensitivity (many tunable parameters)
4. No formal guarantees (empirical metrics only).
5. Interpretability (black-box neural controllers).
6. Model mismatch robustness (parameter drift unanalyzed).
7. Offline training cost (4-5 hours).
8. Distribution mismatch (DeepONet trained on initial conditions).

Practical guidance: Clear recommendations for when to use ROL vs. classical methods vs. pure RL

**Q5:** Consider including other sections and subsections to describe related works

Response: We have added a new "Related Work" section (Section 2) after the Introduction, providing comprehensive coverage:

Subsections include:

1. Neural Operators and Surrogate Modeling: DeepONet, FNO, PINO, PINTO, BIO, VINO.
2. Deep Reinforcement Learning for Chaotic Systems: Recent advances in flow control, KS equation, KAN.
3. Classical and Model-Predictive Control: LQR, backstepping, MPC context.
4. Hybrid and Transfer Learning Approaches: Warm-start initialization, offline RL, HypeRL.
5. Structure-Preserving and Stability-Aware Learning: Lyapunov-based neural networks.
6. Positioning of ROL: Unique position in the landscape.

**REVIEWER #1 COMMENTS:**

**Comment 1:** Lack of relevant references for past 5 years.

**Response:** Addressed under Editor Q1 above. We have added new references from 2024-2025 to bring the state-of-the-art coverage up to date.

**Comment 2:** Research structure needs better organization.

Response:

- Added Related Work section (Section 2) clearly organizing literature
- Added Hyperparameter Tuning subsection (Section 3.9) explaining methodology
- Results now include numerical values with error bars (std, confidence intervals)

**Comment 3:** Add at least three 2025 references.

**Response:** We have added new references from 2025.

**REVIEWER #2 COMMENTS**

**Comment 1:** Consider a separate section for related works.

**Response:** We have added a comprehensive new Related Work section (Section 2) with detailed subsections.

**Comment 2:** Consider numbering for sections and subsections.

**Response:** We have systematically added section numbering throughout the manuscript.

**REVIEWER #3 COMMENTS**

**Comment 1:** Explain more how hyperparameter tuning was performed.

**Response:**  We have added Section 3.9 with detailed grid search documentation for all hyperparameters.

**Comment 2:** Include failure cases and balanced discussion.

We have added:

Section 4.6: Failure Modes and Operating Boundaries (6 scenarios)

Section 4.7: Balanced Assessment (4 strengths + 8 limitations + guidance)

Table 2: Operating envelope with Green/Yellow/Red zones
